# Malaria, Typhoid Fever, and Their Coinfection among Febrile Patients at a Rural Health Center in Northwest Ethiopia: A Cross-Sectional Study

**DOI:** 10.1155/2014/531074

**Published:** 2014-10-01

**Authors:** Meseret Birhanie, Belay Tessema, Getachew Ferede, Mengistu Endris, Bamlaku Enawgaw

**Affiliations:** ^1^Department of Medical Parasitology, School of Biomedical and Laboratory Sciences, College of Medicine and Health Sciences, University of Gondar, P.O. Box 196, Gondar, Ethiopia; ^2^Department of Medical Microbiology, School of Biomedical and Laboratory Sciences, College of Medicine and Health Sciences, University of Gondar, Gondar, Ethiopia; ^3^Department of Hematology & Immunohematology, School of Biomedical and Laboratory Sciences, College of Medicine and Health Sciences, University of Gondar, Gondar, Ethiopia

## Abstract

*Background.* Malaria and typhoid fever are major public health problems in tropical and subtropical countries. People in endemic areas are at risk of contracting both infections concurrently.* Objectives.* The study was aimed at determining the prevalence and associated risk factors of malaria, typhoid, and their coinfection among febrile patients. *Methods.* A cross-sectional study was conducted on 200 febrile patients suspected for malaria and/or typhoid fever from April to May, 2013, at Ayinba Health Center, Northwest Ethiopia. Blood samples were collected for blood culture, Widal test, and blood film preparation. Data were analyzed using SPSS version 20 statistical software.* Results.* The prevalence of malaria was 36.5% (*n* = 73). Among these 32 (43.8%), 30 (41.1%) and 11 (15.1%) were positive for* P. falciparum, P. vivax, *and mixed infections, respectively. The seroprevalence of typhoid fever was 38 (19%), but 1 (0.5%) with blood culture. Malaria typhoid fever coinfection was 13 (6.5%). 2–5-year-old children and poor hand washing habit were significantly associated with malaria and typhoid infection, respectively (*P* < 0.05).* Conclusions.* The prevalence of malaria and typhoid fever was found high. Further studies should be done on the other determinants of malaria and typhoid fever coinfection in different seasons and different study areas.

## 1. Introduction

Malaria is one of the febrile illnesses and the most common fatal disease in the world caused by one or more species of plasmodium. These are* Plasmodium falciparum*,* Plasmodium vivax*,* Plasmodium ovale*,* Plasmodium malariae*, and* Plasmodium knowlesi*. Approximately half of the world population is at risk of malaria. Most of malaria cases and deaths occur in sub-Saharan Africa. According to the World malaria report 2011, there were about 216 million cases of malaria and an estimated 655,000 deaths in 2010 [1].

Malaria is the most communicable disease in Ethiopia and it accounts for about 30% of the overall disability adjusted life years lost. Approximately 68% (54.2 million) of the total population of 78 million lives in malaria risk areas.* P. falciparum* and* P. vivax* are the dominant species of malaria in Ethiopia, with 60% and 40% relative frequencies, respectively.* Plasmodium falciparum* is a predominant species in endemic areas and causes complicated disease and death in the country [2].

Typhoid fever (enteric fever) is a systemic prolonged febrile illness caused by certain Salmonella serotypes.* Salmonella enterica serotype typhi (S. typhi)* and* Salmonella enterica serotype paratyphi (S. paratyphi A, S. paratyphi B,* and* S. paratyphi C)* are species that cause typhoid fever.* S. typhi* is the most common serotype of salmonella that causes typhoid fever [3–6]. The estimated total number of world typhoid fever episode in 2010 was 13.5 million [5]. Poor disposal of human excreta, poorly equipped latrine with water facility, poor hand washing habit, and untreated water usage are the main cause of transmission of typhoid fever in developing countries [4, 5].

Malaria and typhoid fever are a major public health problem in tropical and subtropical countries caused by very different organisms, protozoa and Gram negative bacilli, respectively, and transmitted via different mechanisms [5–8]. People in endemic areas are at a risk of contracting both infections concurrently [9, 10]. There is a considerable overlap of signs and symptoms of malaria and typhoid fever [2, 11–14]. Thus the similarity of clinical features of both diseases leads to misdiagnosis and mistreatment of the febrile patients [11, 15]. So, reliable diagnostic method is important for effective management of cases to reduce misuse and wastage of drugs [11, 14–16]. So far, the prevalence of malaria, typhoid fever, their coinfection, and associated risk factorswere not well studied in Ethiopia. This study was conducted to determine the prevalence of malaria, typhoid fever, and their coinfection among febrile patients.

## 2. Methods

### 2.1. Study Area, Population, and Design

The study was conducted from April 2013 to May 2013 at Ayinba Health Center, Dembia district, Northwest Ethiopia. The altitude of the district ranges between 1,750 and 2,100 m above sea level. It has a population of more than 300,000 and the majority of its population depends on subsistence agriculture. The district is malarious (mainly* P. vivax* and* P. falciparum*) and covers an area of 1,270 Km^2^. It is 27 km away from ancient city Gondar. All febrile patients (age ranged 2–80 years) suspected for malaria and/or typhoid fever who had not taken antimalarial drug and/or antibiotics within 2 weeks were included. Patients with underlying diseases were excluded from the study.

### 2.2. Specimen Collection and Processing

Data on the sociodemographic and clinical characteristics of the study participants were collected using a pretested structured questionnaire by interview. After interview, 10 mL blood sample was collected from adult patients by experienced laboratory technologist. Then, 7 mL blood was inoculated immediately to 45 mL Brain Heart Infusion broth. Similarly, 3-4 mL blood was collected from children and 1.5–2 mL blood was inoculated to 9 mL broth to isolate* S. typhi* and* S. paratyphi*. Both thick and thin blood films were prepared for malaria diagnosis and slide agglutination test was done for typhoid fever screening using somatic (O) and flagellar (H) antigens kits of* S. typhi* (TYDAL, Lab Care Diagnostics (India)). Antibody titration was performed for slide reactive samples. Antibody titer of ≥1 : 80 against O and H antigen of* S. typhi* was taken as a cut of value based on the manufacturer instruction. The blood smear was read at the health center by laboratory technicians and the result was reported. All blood films were reread by experienced microscopist at the University of Gondar Hospital laboratory who was blinded to initial results. Discrepancies occurred in the result by the two readers were solved by using the third experienced microscopist.

### 2.3. Statistical Analysis

The data was cleaned, edited, checked for completeness, entered to Epi Info version 3.5.3, and exported to SPSS version 20 for analysis. Chi-square and odds ratio (OR) by logistic regression were calculated to determine associated factors. *P* value <0.05 was considered statistically significant.

### 2.4. Ethical Consideration

Ethical clearance was obtained from University of Gondar, School of Biomedical and Laboratory Sciences research and ethical committee. Permission was obtained from Dembia Woreda Health Office and Ayinba Health Center. Written informed consent was obtained from each of the volunteer study subjects or guardian of children. Positive results were given for nurses working in the health center for treatment according to the national treatment guideline.

## 3. Results

*Sociodemographic Data*. A total of 200 febrile patients suspected for malaria and/or typhoid fever were included in this study. About 60% of the study participants were males. The mean age was 24.24 ± 13.4 years and majority of the patients (41.5%) were within the age range of 12–25 years, and most of the patients were farmers (71.5%), rural residents (89%), and illiterate (61.5%) (Table 1).

**Figure 1:**
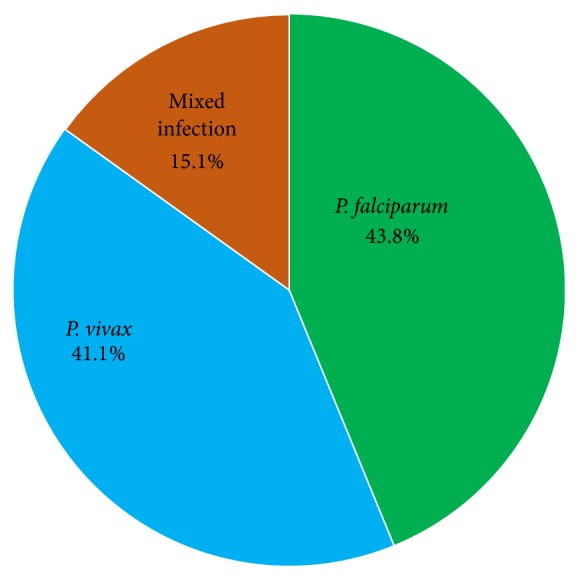
Frequency of malaria among febrile patients at Ayinba Health Center Northwest Ethiopia, April to May 2013.

*Prevalence of Malaria*. Malaria was the most prevalent disease in the study area. From the total 200 febrile patients 73 (36.5%) were malaria positive. Of them, 32 (43.8%) were positive for* P. falciparum,* 30 (41.1%) were positive for* P. vivax, *and the remaining 11 (15.1%) were positive for both* P. falciparum* and* P. vivax. *The positivity rates of* P. falciparum* and* P. vivax* were 51.2% and 48.8%, respectively (Figure 1).

*Prevalence of Typhoid Fever*. Of the total study subjects, 38 (64.4%) patients had antibody titers of ≥1 : 80 for both O and H antigens; of them, 7 (18.4%) had ≥ 1 : 320 titers. Different gram negative organisms were grown on the blood culture but there was only one growth of* S. typhi *(Table 2).

### 3.1. Prevalence of Malaria and Typhoid Fever Coinfection

The titration result showed that the prevalence of coinfection was 13 (6.5%). Of them 8 (61.5%) were coinfected with* P. falciparum, *3 (23.1%) were with* P. vivax,* and 2 (15.4%) were with mixed infection. The prevalence of coinfection using blood culture was 1 (0.5%).

### 3.2. Risk Factors Associated with Malaria, Typhoid Fever, and Their Coinfection

The age group 2–5 years old were significantly associated with malaria (*P* = 0.04). The chi square analyses showed that this age group was significantly associated with malaria (*P* = 0.04). There was no significant association of malaria and typhoid fever with sex, residence, occupation, and educational background (*P* > 0.05) (Table 3). Clinical features were not significantly associated with malaria and typhoid fever (*P* > 0.05) (Table 4).

By using logistic regression analysis, bed net usage, impregnation of the bed net with chemicals, and history of travel to malaria endemic areas were not significantly associated risk factors of malaria (Table 5). But hand washing habit was significantly associated with typhoid fever infection (*P* = 0.01, OR = 2.893, 95% CI = 1.245–6.72) (Table 6).

## 4. Discussion

The result of this study is comparable with the reports from Akoko State, Nigeria, 37.6% [17] and Imo State, Nigeria, 39% [18]. But it is less than the reports from Sierra Leone 62.3% [8] West Gojam, Ethiopia, 62% [19] and Ibadan, Nigeria, 44.3% [20] and higher than the reports in Ebony, Nigeria, 13.2% [21], Enugu, Nigeria, 22.2% [22], Kaduna State, Nigeria, 27% [7], Sokoto, Nigeria, 17% [23], and Benin 5% [24]. The discrepancy of the results between the studies might be due to seasonal variation and difference in geographical locations.

The positivity rates of* P. falciparum* and* P. vivax* were almost similar (51.2% and 48.8%, resp.). But according to the Federal Ministry of Health report the relative frequency of* P. falciparum *and* P. vivax* was 60% and 40%, respectively [2]. There is a great difference in frequencies of two plasmodium species. The difference in the frequencies of the two species might be the result of the prevention and control measures employed in the study area that have higher impact on* P. falciparum* than* P. vivax*. In case of* P. vivax* the dormant stage of the parasite can relapse at any time and relatively maintain its prevalence in the community.

In this study the prevalence of malaria was higher in males 64.4% than females 35.6% but there was no statistically significant association (*P* = 0.337). While other studies showed in Sierra Leone, females (53.4%) are more affected than males (46.6%) [8]. This might be due to the fact that males are sleeping outside their house for agricultural purpose and have greater chance to travel to malaria endemic area for crop cultivation or daily labor. In this study there was significant association between age and malaria (*P* = 0.04). This might be due to low immune response against malaria infection, inappropriate use of bed nets, and in appropriate use of antimalarial drugs in case of children.

The prevalence of the typhoid fever using Widal titration test was comparable with the study in Ebonyi, 21.2% [21], and Ibadan, 16.7% [20], and less than that of the reports in Sierra Leone, 31.4% [8], Kaduna State, 36.6% [7], Akoko, 73.9% [17], Lagos, 27.6% [25], Benin, 39% [24], and Imo State, Nigeria, 42% [18], but higher than the reports from West Gojam 5.8% [19] and Sokoto, Nigeria, 10.3% [23]. This might be due to the differences in Widal test kits, year of study, season, difference in cultural practices, and toilet facility. In addition, the antibody titer levels found in a healthy population may vary from time to time and in different areas, so it is difficult to establish a cut-off level of baseline antibody in a defined area and community [11].

The frequency of typhoid fever was greater in females (22.5%) than males (16.7%), but not statistically significant (*P* = 0.582); however, other previous studies in Sokoto, Nigeria, showed that the frequency of typhoid fever was 29.4% among males and 22.9% among females [23]. Females may acquire infection during food preparation, child care, and other household activities, thus increasing the frequency of typhoid fever.

In this study, the result of malaria and typhoid coinfection using Widal test is comparable with the reports of Ebonyi State 5.6% [21] but higher than the result of study in west Gojam, 2.8% [19] and lower than the reports in Bo city Sierra Leone 14.1% [8], Akoko 18.4% [17], Ibadan 12% [20], Enugu 16% [22], Kaduna 10.1% [7], Sokoto 10.3% [23], and Imo state 22% [18].

The result of the coinfection using blood culture was 0.5% and this is in agreement with the study in Ibadan 0.4% [20], Kaduna 0.5% [7], Sierra Leone 0.6% [8], and Ebony, 0.8% [21] but less than the study in Sokoto, 1.33% [23], Lagos, 19.95% [25], and Enugu, 26.6% [22]. Even though blood culture is a gold standard test for typhoid fever diagnosis, it can be affected by duration of infection, intake of antibiotics [11], and laboratory setup.

Because of the high prevalence of typhoid fever and malaria in the tropics, coinfections are common [9]. The high rate of typhoid and malaria coinfection using Widal test may be responsible for the frequent treatment of mixed infections. However, blood culture results showed that this rate of coinfection is only 0.5%. Hence, typhoid fever could cross-react with malaria using Widal test [6, 11] and lead to overdiagnosis of typhoid fever. Thus, overdiagnosis of typhoid fever leads to unnecessary exposure of patients to the side effects of antibiotics. In addition, misdiagnosis may result in delayed diagnosis and treatment of malaria and other acute febrile illnesses. This emphasized the importance of a reliable diagnostic test for typhoid fever. Study subjects, with poor hand washing habit, were more likely to be affected by typhoid fever (*P* value = 0.04, OR = 2.893, 95% CI = 1.245–6.72).

## 5. Conclusions

Malaria was the most prevalent disease among febrile patients in the study area. There was a substantial result discrepancy among Widal test and blood culture for the diagnosis of typhoid fever. Poor hand washing habit was significantly associated with typhoid fever. Further studies should be done on the other potential risk factors of malaria and typhoid fever coinfection in different seasons and different study areas. The community should be encouraged to use latrine to reduce the burden of high prevalence of typhoid fever infection in the area. The continued development of better diagnostic tools for both malaria and typhoid fever is still crucial.

## Figures and Tables

**Table 1:** Sociodemographic data of study subjects in Ayinba Health Center, Northwest Ethiopia, April to May 2013.

Variables	Frequency	Percentage
Sex		
Male	120	60.0
Female	80	40.0
Age in years		
2–5	12	6.0
6–11	25	12.5
12–25	83	41.5
26–45	66	33.0
≥46	14	7.0
Residence		
Rural	178	89.0
Urban	22	11.0
Education		
Illiterate	123	61.5
Read and write	20	10.0
Primary school	40	22.0
Secondary school	7	3.5
College/university	6	3.0
Occupation		
Farmer	152	76.0
Merchant	7	3.5
Civil servant	3	1.5
Housewife	2	1.0
Daily laborers	4	2.0
Students	32	16.0

**Table 2:** The frequency distribution of titration result among slide agglutination test result in febrile patients at Ayinba Health Center, Northwest Ethiopia, April to May 2013.

Widal titer	Frequency	Percentage
No agglutination	5	8.5
1 : 20	6	10.2
1 : 40	10	16.9
1 : 80	14	23.7
1 : 160	17	28.8
≥1 : 320	7	11.9

Total	59	100.0

≥1 : 80 titer taken as positive titer.

**Table 3:** Prevalence of malaria and typhoid fever and their coinfection in relation to sociodemographic characteristics among febrile patients at Ayinba Health Center, Northwest Ethiopia, from April to May, 2013.

Variables	Malaria				Typhoid fever				Coinfection			
	Positive *N* (%)	Negative *N* (%)	*χ* ^2^	*P* value	Reactive *N* (%)	Nonreactive *N* (%)	*χ* ^2^	*P* value	Positive *N* (%)	Negative *N* (%)	*χ* ^2^	*P* value
Sex												
Male	47 (39.2)	73 (60.8)	0.92	0.337	20 (16.7)	100 (83.3)	1.06	0.30	9 (7.5)	111 (92.5)	0.49	0.482
Female	26 (32.5)	54 (67.5)			18 (22.5)	62 (77.5)			4 (5.0)	6 (95.0)		
Age												
2–5	8 (66.7)	4 (33.3)	10.2	**0.04**	3 (25.0)	9 (75.0)	0.91	0.92	3 (25.0)	9 (75.0)	8.26	0.08
6–11	8 (32.0)	17 (68.0)			6 (24.0)	19 (76.0)			2 (8.0)	23 (92.0)		
12–25	35 (42.2)	48 (57.8)			15 (18.1)	68 (81.9)			5 (6.0)	78 (94.0)		
26–45	20 (30.3)	46 (69.7)			13 (19.7)	53 (80.3)			3 (4.5)	63 (95.5)		
≥46	2 (14.3)	11 (85.7)			2 (14.3)	12 (85.7)			0 (0.0)	14 (100)		
Residence												
Rural	68 (38.2)	110 (61.8)	0.02	0.155	34 (19.1)	144 (80.9)	0.01	0.91	12 (6.7)	166 (93.3)	0.16	0.69
Urban	5 (22.7)	17 (77.3)			4 (18.2)	18 (81.8)			1 (4.5)	21 (95.5)		
Education												
Illiterate	44 (35.8)	79 (64.2)	1.36	0.851	24 (19.5)	99 (80.5)	0.18	0.99	8 (6.5)	115 (93.5)	1.19	0.879
Read and write	8 (40.0)	12 (60.0)			4 (20.8)	16 (80.0)			1 (5.0)	19 (95.0)		
Primary school	17 (38.6)	27 (61.4)			8 (18.2)	36 (81.8)			3 (6.8)	41 (93.2)		
Secondary school	3 (42.9)	4 (57.1)			1 (14.3)	6 (85.7)			1 (14.3)	6 (85.7)		
College	1 (16.7)	5 (83.3)			1 (16.7)	5 (83.3)			0 (0.0)	6 (100)		
Occupation												
Farmer	52 (35.4)	95 (64.6)	8.54	0.20	29 (19.7)	118 (80.3)	2.90	0.82	9 (6.1)	138 (93.8)	2.14	0.906
Merchant	2 (28.6)	5 (71.4)			2 (28.6)	5 (71.4)			1 (14.3)	6 (85.7)		
Civil servant	1 (33.3)	2 (66.7)			0 (0.0)	3 (100.0)			0 (0.0)	3 (100)		
Housewife	0 (0.0)	2 (100.0)			0 (0.0)	2 (100.0)			0 (0.0)	4 (100)		
Daily laborers	0 (0.0)	4 (100.0)			1 (25.0)	3 (75.0)			0 (0.0)	2 (100)		
Student	14 (43.8)	18 (56.2)			6 (18.8)	26 (81.2)			3 (9.4)	29 (90.6)		

*N*: number; *χ* ^2^: Chi square.

**Table 4:** Prevalence of malaria and typhoid fever infection in relation to clinical features in febrile patients at Ayinba Health Center, Northwest Ethiopia, from April to May 2013.

Clinical features	Malaria				Typhoid fever				Coinfection			
	Positive *N* (%)	Negative *N* (%)	*χ* ^2^	*P* value	Reactive *N* (%)	Nonreactive *N* (%)	*χ* ^2^	*P* value	Positive *N* (%)	Negative *N* (%)	*χ* ^2^	*P* value
Fever												
Continuous	27 (34.2)	52 (65.8)	0.30	0.58	15 (19)	64 (81)	0.00	0.99	4 (5.1)	75 (94.9)	0.44	0.5
Intermittent	46 (38.0)	75 (62.0)			23 (19)	98 (81)			9 (7.4)	112 (92.6		
Headache												
Yes	66 (36.1)	117 (63.9)	0.18	0.68	34 (18.6)	149 (81.4)	0.24	0.62	10 (5.4)	174 (94.6)	4.29	0.06
No	7 (41.2)	10 (58.8)			4 (23.5)	13 (76.5)			3 (16.7)	13 (83.3)		
Joint pain												
Yes	55 (36.2)	97 (63.8)	0.03	0.87	28 (18.4)	124 (81.6)	0.14	0.71	8 (5.3)	144 (94.5)	1.59	0.44
No	18 (37.5)	30 (65.5)			10 (20.8)	38 (79.2)			5 (10.4)	43 (89.6)		
Vomiting												
Yes	33 (38.8)	52 (61.2)	0.34	0.56	13 (15.3)	72 (84.7)	1.32	0.25	4 (4.7)	81 (95.3)	0.78	0.38
No	40 (34.8)	75 (65.2)			25 (21.7)	90 (78.3)			9 (7.8)	106 (92.2)		
Chill/rigor												
Yes	63 (38.0)	103 (62.0)	0.89	0.35	29 (17.5)	137 (82.5)	1.48	0.22	10 (6.0)	156 (94.0)	0.36	0.55
No	10 (29.4)	24 (70.6)			9 (26.5)	25 (73.5)			3 (8.8)	31 (91.2)		
Fatigue												
Yes	70 (38.5)	112 (61.5)	3.36	0.07	33 (18.1)	149 (81.9)	0.99	0.32	12 (6.6)	170 (93.4)	0.03	0.86
No	3 (16.7)	15 (83.3)			5 (27.8)	13 (72.2)			1 (5.6)	17 (94.4)		
Nausea												
Yes	49 (36.6)	85 (63.4)	0.00	0.98	29 (21.6)	105 (78.4)	1.84	0.18	9 (6.7)	125 (93.3)	0.03	0.86
No	24 (36.4)	42 (63.6)			9 (13.6)	57 (86.4)			4 (6.1)	62 (93.9)		
Diarrhea												
Yes	27 (37.0)	46 (63.0)	0.01	0.91	19 (25.7)	55 (74.3)	3.40	0.06	6 (8.2)	67 (91.8)	0.56	0.45
No	46 (36.2)	81 (63.8)			19 (15.1)	107 (84.9)			7 (5.5)	120 (94.5)		
Constipation												
Yes	14 (40)	21 (60)	0.05	0.83	6 (17.1)	29 (82.9)	0.49	0.82	4 (11.8)	30 (88.2)	1.87	0.17
No	59 (35.8)	106 (64.2)			32 (19.4)	133 (80.6)			9 (5.4)	157 (94.6)		

*N*: number; *χ* ^2^: Chi square.

**Table 5:** Determinants associated with malaria in febrile patients at Ayinba Health Center, Northwest Ethiopia, from April to May 2013.

Variables	Positive *N* (%)	Negative *N* (%)	*P* value	COR	95% CI
History of travel					
Yes	10 (37.0)	17 (63.0)	0.95	0.974	0.420–2.256
No	63 (36.4)	110 (63.6)		1	
Bed net usage					
Yes	56 (38.9)	88 (61.1)		1	
No	17 (30.4)	39 (69.9)	0.26	1.685	0.354–1.326
Impregnation of bed net					
Yes	3 (18.8)	13 (81.2)		1	
No	53 (41.4)	75 (58.6)	0.366	1.889	0.476–7.497

*N*: number; COR: crude odds ratio; CI: confidence interval.

**Table 6:** Determinants associated with typhoid fever in febrile patients at Ayinba Health Center, Northwest Ethiopia, from April to May 2013.

Variables	Typhoid fever				
	Reactive *N* (%)	Nonreactive *N* (%)	*P* value	COR	95% CI
Water source					
Tap water	13 (23.2)	43 (76.8)		1	
River	6 (27.2)	16 (72.7)	0.77	1.24	0.403–3.820
Spring	17 (16.0)	89 (84.0)	0.26	0.632	0.281–1.418
Well	2 (12.5)	14 (87.5)	0.36	0.473	0.095–2.235
Uncooked food feeding					
Yes	14 (21.2)	52 (78.8)	0.57	1.234	0.590–2.57
No	24 (17.9)	110 (82.1)		1	
Toilet usage					
Yes	34 (19.1)	144 (80.9)		1	
No	4 (18.2)	18 (81.8)	0.91	0.941	0.299–2.961
Hand washing habit					
Poor	11 (35.5)	20 (64.5)	**0.01**	**2.893**	**1.245–6.720**
Good	27 (16.0)	142 (84.0)		1	

*N*: number; COR: crude odds ratio; CI: confidence interval.
